# Optimising Hydrocarbon Extraction from Soil Using Mixed-Surfactant Systems

**DOI:** 10.3390/toxics14020153

**Published:** 2026-02-03

**Authors:** Emilio Ritoré, Carmen Arnaiz, José Morillo, Agata Egea-Corbacho, José Usero

**Affiliations:** Department of Chemical and Environmental Engineering, Higher Technical School of Engineering, University of Seville, Camino de los Descubrimientos s/n, 41092 Seville, Spain; ese_emi@hotmail.com (E.R.); jmorillo@us.es (J.M.); aegeacorbacho@us.es (A.E.-C.); usero@us.es (J.U.)

**Keywords:** hydrocarbons, petroleum, soil remediation, soil flushing, hydrocarbons fractions

## Abstract

In industrial settings, one of the key environmental challenges is the remediation of soil contaminated by hydrocarbons. Washing the soil with surfactants mobilises and extracts these compounds, making them easier to treat. As it enables the recovery and reuse of soil within sustainable production processes, this technique is part of the circular economy. Soil-washing experiments using surfactants were carried out to determine whether a mixture of anionic and non-ionic surfactants could improve the remediation of soil contaminated by gasoline and diesel fuel compared to the use of a single surfactant. Four surfactants were used (non-ionic: polyoxyethylene lauryl ether and polyoxyethylene (80) sorbitan monooleate; anionic: sodium dodecylbenzenesulfonate and sodium dodecyl sulfate). The aliphatic and aromatic hydrocarbon fractions (C6–C8, C8–C10, C10–C12, C12–C16, C16–C21 and C21–C35) of gasoline and diesel fuel were analysed. Sodium dodecylbenzenesulfonate was selected for the purpose of preparing mixtures with the other two non-ionic surfactants, polyoxyethylene lauryl ether and polyoxyethylene (80) sorbitan monooleate. These surfactant mixtures demonstrated significantly higher removal rates than sodium dodecylbenzenesulfonate alone. Mixtures of sodium dodecylbenzenesulfonate and polyoxyethylene lauryl ether achieved hydrocarbon extraction of between 61% and 68%, while sodium dodecylbenzenesulfonate-polyoxyethylene (80) sorbitan monooleate mixtures obtained extraction of between 58% and 66%. Analysis of the gasoline and diesel hydrocarbon fractions indicated that smaller molecules desorb more easily than larger ones and that aromatics desorb more easily than aliphatics. Furthermore, the mixtures increased the extraction of both aliphatic and aromatic hydrocarbons, particularly the lighter compounds. The variation on removal rates within the hydrocarbon ranges may be related to the octanol–water partition coefficient (K_ow_). These improvements with mixtures of anionic and non-ionic surfactants could be exploited to enhance the effectiveness of surfactant-flushing treatments and optimise the design of soil surfactant treatments.

## 1. Introduction

Gasoline and diesel fuel are multicomponent products resulting from petroleum refining processes, mainly composed of hydrophobic organic compounds [1]. These fuels are widely used in the transport industry for vehicles, ships, trains and trucks. The contamination of groundwater and soil by gasoline and diesel is predominantly attributable to transportation-related incidents and structural failures in underground storage tanks [2].

Most spills occur either in marine environments due to tanker accidents or on land through leaks from pipelines and storage systems. Among terrestrial sources, fuel stations and their underground storage tanks represent a major cause of soil and groundwater contamination [3]. These constitute recurrent sources of petroleum release into the subsurface environment [4,5]. For this reason, European regulations have promoted the upgrading of these tanks to minimise the risk of fuel leakage [6].

The removal of hydrocarbons present in water can be addressed using various remediation techniques. Currently, the available methods fall into four broad categories: biological, physical, thermal, and chemical remediation. Together, these strategies seek to remove petroleum-derived contaminants while minimising disruption to the soil matrix and preserving its original properties as much as possible [7,8]. Physical–chemical treatments encompass a broad set of techniques that rely on physical processes, chemical or electrochemical reactions, thermal and heat-based operations, as well as acoustic and ultrasonic methods. In contrast, biological treatments depend on natural mechanisms driven by microorganisms or plants to degrade or immobilise contaminants. Overall, physical–chemical approaches tend to offer rapid and efficient remediation, although many of them are costly and may limit the future use of the soil. Biological treatments are generally more environmentally friendly, but they often require longer periods to achieve effective contaminant removal [9].

The selection of an appropriate and effective treatment for soils impacted by gasoline or diesel depends on the physicochemical properties of the soil and the pollutants, as well as on economic considerations and the time required for implementation [10]. Common methods include soil vapour extraction [11,12], pump-and-treat [13,14], thermal treatment [15], surfactant soil flushing [16,17], in situ chemical oxidation [18], and bioremediation [19,20], electrokinetics and photocatalysis [21,22].

Atemoagbo [23] presents a review showing that biological methods such as biodegradation and phytoremediation can achieve high contaminant removal rates (85–95%) at relatively low costs (50–100 USD/m^3^). However, the analysis also highlights that these technologies often require long treatment periods and are strongly dependent on environmental conditions, which limits their applicability in scenarios where rapid intervention or precise process control is needed. In contrast, physicochemical techniques, including chemical oxidation and solvent extraction, typically reach efficiencies of 60–80% with more variable costs (100–500 USD/m^3^), but they offer much shorter treatment times and greater adaptability to different soil types and contaminants.

In the context of sustainability, the recent literature highlights that the different soil remediation strategies present clearly differentiated environmental, operational, and economic implications. While physical and physicochemical methods often achieve rapid reductions in contaminant concentrations, their application involves high energy consumption, secondary waste generation, and greater operational costs, which limits their long-term sustainability. In contrast, biological techniques, such as biopiles or biostimulation, rely on autochthonous microorganisms capable of efficiently degrading hydrocarbons under controlled conditions, reducing environmental impacts and promoting more circular processes with a lower ecological footprint [24]. The study by Micle and Sur [25] shows that through appropriate design of aeration, moisture, and microbial amendments, it is possible to achieve remediation efficiencies of 83% in just 12 weeks, demonstrating that biological technologies are not only viable but also more consistent with the principles of sustainability and the circular economy in the management of contaminated soils.

However, according to the possible treatments and their selection, it should be noted that after a spill, hydrophobic organic compounds are absorbed by the soil due to interactions between soil components and hydrocarbon molecules [26]. Furthermore, hydrocarbons cannot be removed by groundwater extraction due to their low solubility in water [27]. One remediation method that addresses these problems is soil washing with surfactants. Surfactant solutions reduce the interfacial tension between hydrocarbons and soil components [28]. In addition, the solubility of hydrocarbons increases significantly once the critical micelle concentration (CMC) is reached [29]. Another factor that must be considered is that surfactants and biosurfactants have the capacity to improve desorption by reducing interfacial tension, thereby increasing the accessibility of hydrocarbons to degrading microorganisms [30,31]. For this reason, in specific scenarios, surfactant-enhanced soil washing constitutes an appropriate remediation strategy, as it obviates the need for excavation, minimises disturbance to the soil matrix, and reduces occupational exposure to hazardous substances [32,33].

Studies conducted over the past decades have confirmed that applying a surfactant is an effective way of treating soils contaminated with gasoline or diesel. Baziar et al. [34] achieved diesel removal efficiencies of 80% and 65% using polyoxyethylene (80) sorbitan monooleate and polyoxyethylene lauryl ether, respectively. Deshpande et al. [35] and Khalladi et al. [36], meanwhile, used anionic surfactants for the removal of gasoline and diesel fuels. López et al. [37] and Zhu et al. [38] demonstrated that total petroleum hydrocarbon (TPH) desorption levels of up to 60% could be achieved using non-ionic surfactants. Xu et al. [39] have reported that in situ remediation using surfactants can cost around 90 USD per ton of soil, whereas ex situ applications may reach up to 150 USD per ton.

Therefore, it can be concluded that the efficiency of hydrocarbon removal using a single surfactant is limited. A promising solution is to use surfactant mixtures. On the one hand, anionic surfactants are less sorbed by soil than non-ionic surfactants due to electrostatic repulsion produced by the negative charge of soil components and anionic surfactants [40]. Conversely, the presence of non-ionic surfactants could reduce precipitation between anionic surfactants and soil cations [39,41]. Consequently, mixed anionic–non-ionic surfactants could enhance the efficiency of soil-flushing remediation processes.

Previous studies have investigated the solubilisation of chlorinated organic compounds (COCs) and polycyclic aromatic hydrocarbons (PAHs) using mixtures of anionic and non-ionic surfactants. These studies have demonstrated the effectiveness of mixed surfactants [41,42,43,44,45,46]. Other authors have also investigated the desorption of COCs and PAHs from soil using mixed surfactants, achieving promising results [42,47,48,49,50,51]. However, the desorption of hydrocarbons from polluted soils using gasoline and diesel in conjunction with mixed surfactants has scarcely been investigated.

Investigating the desorption rates of different hydrocarbon fractions is essential. PAHs are a widespread class of carcinogenic aromatic hydrocarbon pollutant [52], and they are highly hydrophobic, low-volatility compounds with a high sorption capacity, particularly the long-chain or heavy-molecular-weight varieties [53]. Conversely, aliphatic hydrocarbons comprise biomarkers such as hopanes and steranes [54]. Analysing these could help to identify spilled fuels and provide additional information on the source of contamination and the degradation of the spill.

The aim of this study is twofold: (i) to determine whether a mixture of anionic and non-ionic surfactants can improve the remediation of gasoline- and diesel-contaminated soil, and (ii) to establish whether preferential desorption occurs between different gasoline and diesel hydrocarbon compounds.

Although previous studies have demonstrated the effectiveness of using a mixture of anionic and non-ionic surfactants to remove soil pollutants such as naphthalene, anthracene and acetone, no studies have examined the removal of the wide range of hydrocarbons found in gasoline and diesel. The main novelties of this research are: (1) to evaluate surfactant mixtures and their combined effects on the remediation of soils contaminated with a gasoline–diesel mixture; and (2) to provide a detailed analysis of the composition of the hydrocarbons. Previous studies of petroleum hydrocarbons only examined the efficacy of surfactants for total petroleum hydrocarbons (TPHs), whereas the present study examined different ranges of aliphatic and aromatic hydrocarbons (C6–C8, C8–C10, C10–C12, C12–C16, C16–C21 and C21–C35) in a gasoline–diesel fuel mixture. Consequently, fractional removal rates can be obtained instead of overall removal rates. Investigating the desorption rates of different types of hydrocarbons is important because gasoline and diesel fuel contain both easily removable and recalcitrant compounds.

## 2. Materials and Methods

### 2.1. Soil Sampling and Contaminants

Non-contaminated soil was collected from Los Marines (Huelva, Spain). The soil samples were taken at a depth of 10–40 cm. Samples were obtained from a depth interval of 10–40 cm. The soil was gently disaggregated and passed through a 0.5 mm sieve. Prior to sieving, the material was air-dried and thoroughly mixed to ensure sample homogenisation. The physicochemical properties of the soil are summarised in Table 1. Analyses included texture, soil organic matter content, and pH.

Subsequently, a mixture of gasoline and diesel fuel (60:40, *v*/*v*) was blended with the Los Marines soil. The fuel mixture was added gradually to ensure uniform distribution throughout the matrix. TPH (C6–C35) were quantified using gas chromatography coupled with mass spectrometry (GC–MS). Table 2 summarises the concentrations determined for each hydrocarbon fraction, as well as the initial TPH content of the contaminated soil (8530 mg kg^−1^).

The soil hydrocarbons were analysed by GC–MS, and the analysis was performed in triplicate. The amount of soil hydrocarbons removed was calculated from the difference of the initial and final concentrations after washing with the surfactant solution. Hydrocarbon analysis was performed as described by Ritoré et al. [24,40]. Data obtained from the laboratory were analysed by SPSS (version 21) using the *t* test and the analysis of variance.

### 2.2. Surfactant

Polyoxyethylene lauryl ether (Brij 35), polyoxyethylene (80) sorbitan monooleate (Tween 80) and sodium dodecyl benzenesulfonate (SDBS) were supplied by Sigma Aldrich (Barcelona, Spain). Sodium dodecyl sulfate (SDS) was obtained from Panreac Applichem (Barcelona, Spain). All of these are analytical-grade. Brij 35 and Tween 80 are non-ionic surfactants with a CMC of 0.09 mmol/L [34] and 0.01 mmol/L [55], respectively. However, SDBS and SDS are anionic surfactants with a higher CMC, 2.76 mmol/L [56] and 8.2 mmol/L [57], respectively. These surfactants were selected because previous studies showed their great solubilisation capacity [36,55], high biodegradability and low human toxicity [58]. Surfactants were dissolved in ultrapure water.

### 2.3. Experimental Procedure

Spiked soil samples (200 g) were placed in 2 L glass bottles, which were then sealed with Teflon^®^ screw caps supplied by Scharlab (Barcelona, Spain) and stored in the dark for 14 days. After this equilibration period, 1.6 L of surfactant solution were added to each bottle, resulting in a solution-to-soil ratio of 8:1. The bottles were capped again and shaken, and the soil–solution mixture was allowed to stand for 24 h. A control experiment was conducted using ultrapure water instead of the surfactant solutions.

The optimal surfactant concentration was defined as the level that provided the most efficient balance between the amount of surfactant applied and the corresponding removal of hydrocarbons from the soil. Three mass-to-volume concentrations were evaluated: 0.5%, 1.5%, and 3.5%. The concentrations identified as optimal in these preliminary assays were subsequently employed in the experiments involving surfactant mixtures.

It is important to note that surfactants may undergo partial adsorption onto the soil solid matrix, which can reduce their effective concentration in solution and influence desorption efficiency. Despite the absence of experimental quantification of this effect in the present study, the process is well documented in the context of surfactant-enhanced remediation [59].

## 3. Results and Discussion

### 3.1. Effect of Surfactant Concentration on the Hydrocarbon Removal Rate

**Figure 1:**
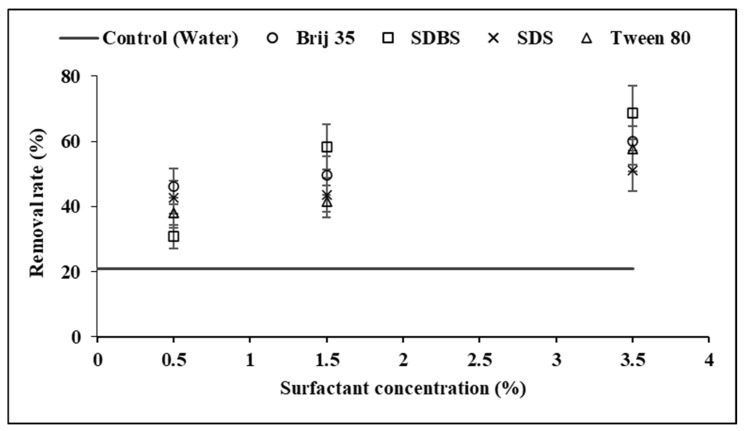
Effect of surfactant concentration on TPH removal rate.

Prior to analysing the surfactant mixtures, a series of laboratory tests was conducted to determine the most suitable concentration for removing oil hydrocarbons from contaminated soil samples. Figure 1 shows the ability of the surfactants to remove TPH (C6–C35) from the soil. Removal efficiencies were evaluated at three solution concentrations for each of the four selected surfactants: 0.5, 1.5, and 3.5% [mass (g)/water volume (mL)].

The results showed that hydrocarbon removal was limited in the blank (water), whereas the surfactant solutions increased the removal rates of gasoline and diesel range hydrocarbons from the soil. As shown in Figure 1, water alone removed 21% of the initial hydrocarbons. This finding is consistent with other authors [36,60], who reported similar petroleum hydrocarbon desorption rates using water in column experiments. In general, petroleum hydrocarbon desorption increased as surfactant concentration rose. The highest removal efficiency was achieved with the anionic surfactant SDBS, which reached 69% hydrocarbon removal at a concentration of 3.5% (Figure 1). At 1.5%, SDBS exhibited between 13% and 28% higher desorption than the other three surfactants tested. At the lowest concentration (0.5%), SDBS removed 31% of petroleum hydrocarbons, whereas at 1.5% and 3.5% it achieved removal rates exceeding 58%. This behaviour may be attributed to the larger micelle core radius at higher concentrations, which can enhance the solubilisation of petroleum hydrocarbons. These results are consistent with those reported by other authors [61]. The non-ionic surfactant Brij 35 slightly increased the desorption of hydrocarbons from the soil as the surfactant concentration rose. The removal efficiency was 46, 50 and 60% with Brij 35 concentrations of 0.5, 1.5 and 3.5%, respectively (Figure 1).

Maximum removal rates of 51% and 58% were achieved when higher concentrations of SDS and Tween 80 were used, respectively (Figure 1). The results shown in Figure 1 are consistent with those reported by Ceschia et al. [57], who found that a 1% SDS solution removed 40% of crude oil hydrocarbons from contaminated soil. However, these findings differ somewhat from those of López et al. [37], who reported hydrocarbon removal efficiencies of 69.4% with Tween 80 and 56% with SDS. These discrepancies may be attributed to the smaller experimental scale used in their study, the higher initial hydrocarbon concentration in the soil, and differences in soil properties.

Khalladi et al. [36] also reported more than 70% diesel removal using SDS in a soil column experiment, although this was achieved with a much higher surfactant-solution-volume-to-soil-mass ratio (83:1). Hernández-Espriú et al. [62] removed diesel from soil using a 0.5% SDS solution (60% removal), a 0.5% Brij 35 solution (41% removal), and a 1% Tween 80 solution (33% removal). The results obtained by Hernández-Espriú et al. [62] for Brij 35 and Tween 80 are comparable to those illustrated in Figure 1.

In line with these findings, recent research on the remediation of real marine sediments has also demonstrated the effectiveness of surfactant-enhanced washing for improving TPH removal. Russo et al. [61] reported that individual surfactants such as SDBS and Tween 80 substantially increased hydrocarbon extraction compared to water alone, achieving removal efficiencies of 42% and 44%, respectively, at low concentrations. Notably, their study showed that combining anionic and non-ionic surfactants further enhanced performance, reaching up to 70% TPH removal.

Tween 80 significantly increased the hydrocarbon removal with a rise in surfactant concentration. At the lowest concentration (0.5%), this non-ionic surfactant desorbed 38% of the petroleum hydrocarbons, but at a surfactant concentration of 3.5% it reached a 58% removal rate (Figure 1). This phenomenon could be explained by the adsorption that the soil gives to surfactants. At low concentrations, Tween 80 monomers are mostly adsorbed by the soil. In this case, the monomers cannot form micelles that solubilise hydrophobic organic compounds, but when the surfactant concentration increases, monomers may create micelles, and they rapidly raise the solubilisation of contaminants [63]. Tween 80, due to its low CMC, attained a significant enhancement in petroleum hydrocarbon desorption. Jousse et al. [64] polluted the soil with n-decane as a representative contaminant of the petroleum hydrocarbons in their study but obtained lower desorption percentages with Tween 80 than those shown in Figure 1. They managed to eliminate 8% of n-decane, perhaps due to the surfactant concentration used (0.0157%). Deshpande et al. [35] analysed the behaviour of anionic and non-ionic surfactants in contaminated soils by hydrophobic organic compounds, and they emphasised that soil surfactant sorption is a very important factor, especially for non-ionic surfactant soil washing. This sorption reduces the potential solubilisation of surfactants.

Considering the data reported above, the surfactant concentration used in the subsequent experiments was 1.5% of SDBS, since the hydrocarbon desorption increase obtained at 3.5% concentration was relatively insignificant, considering that surfactant consumption was more than double. These results are consistent with Singh & John [65], who determined that the best concentration of SDBS to remediate two soils polluted with gasoline was between 1 and 1.5%.

With regard to toxicity, non-ionic surfactants such as Tween 80 and Brij 35 exhibit very low toxicity towards soil microorganisms and, in many cases, may even enhance biodegradation by increasing the solubility and availability of hydrocarbons [66,67]. Their tendency to adsorb partially onto the soil matrix further reduces their direct impact on microbial communities. High concentrations of Brij 35, in contrast to Tween 80, may exert stronger effects on microbial activity [68]. However, despite being generally considered non-toxic—for example, Tween 80, which is widely used as an additive in foods, pharmaceutical formulations, and cosmetics as an emulsifier, dispersant, or stabiliser [69]—it is important to consider the applied concentrations, as these compounds may exert inhibitory effects at levels higher than those typically used in PAH-contaminated soil remediation [70,71]. Tween 80 did not exhibit toxic effects on the microorganisms until its concentration reached 2000 mg L^−1^, whereas Brij 30 inhibited microbial growth at concentrations above 1000 mg L^−1^. In addition, anionic surfactants were observed to be more toxic to bacterial growth than non-ionic surfactants [72].

Anionic surfactants such as SDS and SDBS exhibit a higher inhibitory potential due to their ability to interact with cell membranes and affect sensitive microbial populations, particularly when present at elevated concentrations or when they persist in the environment [73,74]. Liu et al. [72] reported that at the same surfactant concentration, the inhibitory effects of anionic surfactants (SDS and SDBS) on microbial growth and PAH degradation were stronger than those of non-ionic surfactants (Tween 80, Triton X-100, and Brij 30).

Consequently, soil-washing and soil-flushing processes frequently incorporate a concluding rinse with purified water following surfactant application, and they may also include steps for surfactant recovery and reuse to improve the sustainability and cost-efficiency of the treatment [75]. This is done to decrease residual surfactant levels and mitigate potential impacts on microbial activity, particularly when a subsequent bioremediation stage is scheduled.

### 3.2. Efficiency of Mixed-Surfactant Formulations in TPH Removal

The surfactant SDBS, which achieved the best performance in the previous tests (Section 3.1) at a concentration of 1.5 wt/vol%, was subsequently mixed with two non-ionic surfactants (Brij 35 and Tween 80) in different proportions. The main objective was to evaluate whether the surfactant mixtures enhanced the desorption of petroleum hydrocarbons compared to the use of SDBS alone. Table 3 presents the proportions of each surfactant used in the mixtures for the different samples.

**Figure 2:**
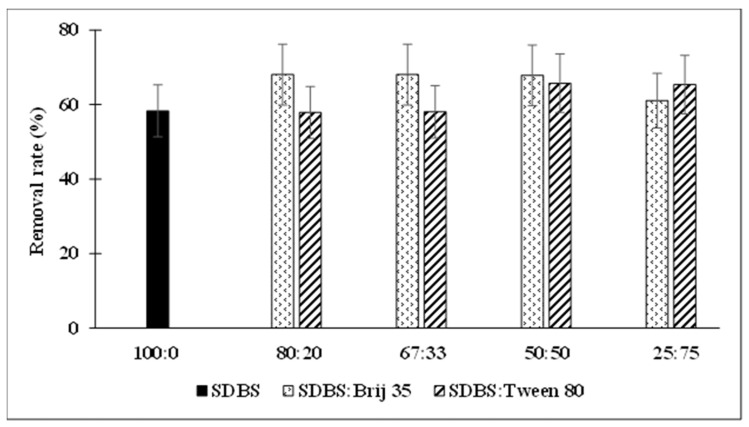
Mixed surfactant and TPH desorption rates.

Figure 2 shows the TPH (C6–C35) removal rates obtained with the surfactant mixtures compared to the removal achieved by SDBS alone at the same concentration and under identical soil conditions. Overall, the mixed-surfactant systems exhibited higher hydrocarbon desorption than SDBS used individually (Figure 2). The mixture that produced the best performance was SDBS combined with Brij 35, reaching removal efficiencies of approximately 68%, except for the 25:75 ratio, where TPH desorption decreased to 61%. Increasing the proportion of Brij 35 relative to SDBS led to a decline in hydrocarbon removal. Nevertheless, all SDBS–Brij 35 mixtures achieved substantially higher desorption than SDBS alone, which removed 58% TPH.

The SDBS Tween 80 mixtures also showed improved removal as the proportion of Tween 80 increased (Figure 2), stabilising at the 50:50 and 25:75 ratios, where hydrocarbon removal approached 66%. When the proportion of Tween 80 was lower (80:20 and 67:33), the results suggested that Tween 80 did not enhance solubilisation within the mixture, as the removal efficiencies were similar to those obtained with SDBS alone (58%).

Numerous studies have been carried out with a mixed surfactant on soils contaminated with PAHs. Yang et al. [49] studied the effect of a mixed surfactant, octylphenol polyethoxylate (TX-100) and SDBS, on phenanthrene desorption, and they demonstrated that mixed surfactant had greater efficiency than individual surfactants. The same results were reported by Zhou & Zhu [50,51], but they used other mixed-surfactant solutions formed by SDS and TX-100 for desorbing phenanthrene from the soil. Also, Shi et al. [48] investigated the removal of 16 PAHs with TX-100 and SDS mixed-surfactant solutions, and they achieved a maximum desorption of 63.6% with a 9:1 ratio (TX-100:SDS). Zhang & Zhu [41] achieved great desorption rates with SDBS-Tween 80, increasing PAH removal. Sales & Fernández [47] analysed the effect of non-ionic surfactants Tween 80 and an anionic one, sodium laurate, creating mixed micelles; the mixture showed an adsorption reduction in the soil with respect to a single surfactant and increased PAH (naphthalene and phenanthrene) desorption. All of them obtained better desorption rates with surfactant mixtures than with individual surfactants. These similar results could be attributed to the anionic surfactants in the mixture assisting in a lower adsorption of surfactants by the soil. For this reason, more surfactant monomers in solutions are available to form micelles that could solubilise hydrophobic organic compounds. Furthermore, non-ionic surfactants decrease the critical micelle concentration over the same amount of surfactant added [76]. This phenomenon occurs because the hydrophobic tails of the anionic surfactant monomers could become embedded in the micelles of non-ionic surfactants, forming a greater number of smaller micelles [55]. In addition, in the presence of the non-ionic surfactant, the precipitation of the anionic surfactant caused by the cations of the soil is reduced.

Despite previously reported studies, few studies on the soil desorption of gasoline or diesel fuel with mixed surfactants, and especially emphasising the hydrocarbons and their chains, have been published. The results in Figure 2 agree with Zhu et al. [38], who assessed several surfactants to remedy soil contaminated with diesel oil. They showed that a mixture of anionic surfactants (sodium alcohol polyethoxylated ether sulfate, AES) and non-ionic surfactants (aliphatic polyethenoxy ether, AEO9) could increase the hydrocarbon removal rate from soil in relation to the use of individual surfactants. A 1% solution of this surfactant mixture was used to remove 60% of soil petroleum hydrocarbons. The petroleum removal increases by surfactant mixtures were 10 and 20% with respect to the use of AEO9 or AES alone, respectively.

Although the soil used was classified as silt loam (20.6% sand, 58.2% silt, 21.2% clay) with moderate organic matter content (5.1%) and slightly acidic pH (5.9) (Table 1), it still represents only one specific soil condition. Therefore, the results may not fully capture the variability found in field soils with contrasting textures, mineralogy, and organic matter levels.

### 3.3. Mixed Surfactants: Hydrocarbon Fractions

The surfactant-flushing soil remediation studies sometimes analysed the different fractions of petroleum hydrocarbons [77,78,79,80,81]. However, very few studies have examined the behaviour of the different hydrocarbon fractions in gasoline- and diesel-contaminated soils treated with mixed surfactants. Most research has focused primarily on the overall TPH response. Gasoline and diesel fuel have compounds that are recalcitrant and others which are easy to desorb; for this reason, it is crucial to examine the removal of petroleum hydrocarbon fractions. There are hundreds of hydrocarbons in gasoline and diesel. In this study, they are clustered into aliphatic and aromatic in order of their size (number of carbon atoms).

**Figure 3:**
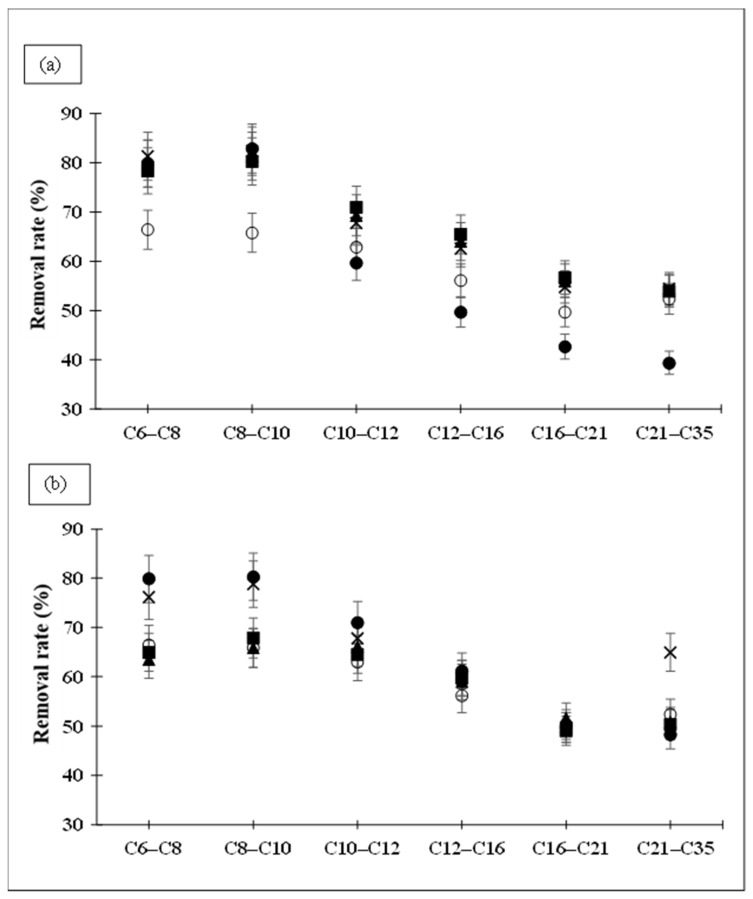
Effect of surfactant mixtures (**a**) SDBS-Brij 35 and (**b**) SDBS-Tween 80 on the hydrocarbon removal rate according to their size (○ 100:0%, ■ 80:20%, ▲ 67:33%, × 50:50%, ● 25:75%).

Figure 3 shows the desorption rates of hydrocarbons according to size. The desorption produced by the surfactant-mixture solutions is indicated. The decrease in desorption as the size of hydrocarbon compounds rose is confirmed by Figure 3. For all mixtures and proportions, hydrophobic organic compound removal rates were lower for higher hydrocarbon ranges compared to smaller fractions. Surfactant mixtures showed a constant decrease in hydrocarbon extraction as the hydrocarbon range increased (Figure 3a,b). The highest removal was reached in the C6–C8 fraction, 70–80%, and it gradually decreased to around 50% for the C21–C35 fraction. Surfactant mixtures produced greater increases in the desorption of smaller-sized compounds than larger ones compared to SDBS alone. SDBS-Brij 35 mixtures (80-20, 67-33 and 50-50 ratio) increased the desorption of all hydrocarbon sizes, although the C21–C35 fraction was limited to 2% higher than SDBS (Figure 3a). On the other hand, the SDBS-Tween 80 mixtures in the proportions 80-20 and 67-33 obtained very similar removal rates in all hydrocarbon sizes with the SDBS solution, but for the proportions in which the amount of Tween 80 was increased (50-50 and 25-75), there were significant increases in the extraction of hydrocarbons smaller than C10. The results in Figure 3 seem to be accurate because the increase in solubilisation has a greater effect on the most soluble compounds, the lighter compounds. This improvement in efficacy concerns compounds that were previously better removed with the use of SDBS alone. The mixtures do not appear to enhance the removal rates of the most difficult hydrocarbons to desorb from the soil.

**Figure 4:**
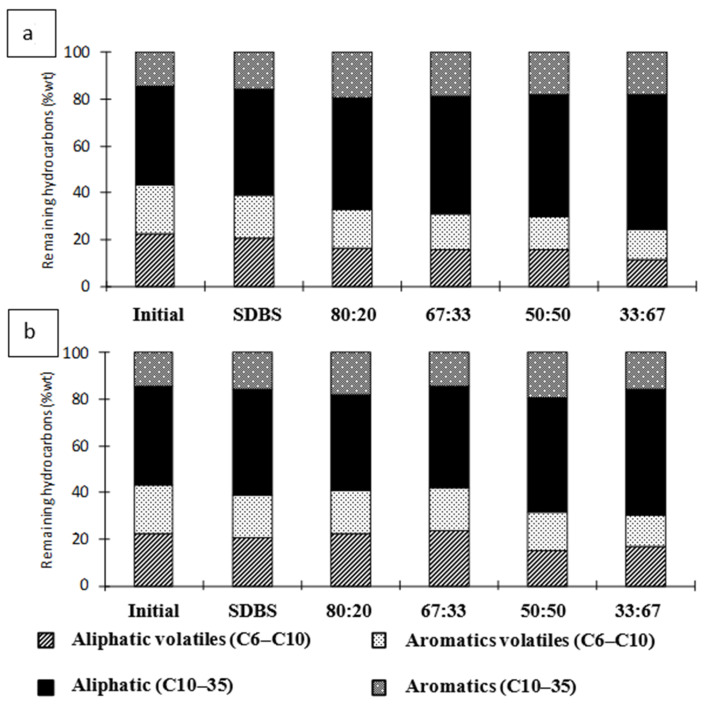
Remaining hydrocarbon ranges in the soil after treatment with mixed surfactants (**a**) SDBS-Brij 35 and (**b**) SDBS-Tween 80 expressed as percentage by weight, %wt.

Figure 4 shows the differences in the removal of aliphatic and aromatic compounds, classified according to their carbon-chain size. Overall, aromatic hydrocarbons were removed more effectively than their aliphatic counterparts.

Table 4 shows that the decrease in removal efficiency with increasing hydrocarbon size, as also illustrated in Figure 3, was observed for both aromatic and aliphatic groups. SDBS extracted slightly more aromatic than aliphatic compounds (61% and 57%, respectively), with the largest differences occurring in hydrocarbons containing fewer than 16 carbons.

The SDBS–Brij 35 mixtures increased removal efficiencies relative to SDBS alone for both aromatic and aliphatic hydrocarbons, with comparable improvements in both groups. The 80:20 SDBS–Brij 35 mixture removed identical proportions of aliphatic and aromatic compounds (68%). However, as the proportion of Brij 35 increased (25:75), the difference between groups widened, with higher extraction of aromatics. In the 25:75 mixture, aromatic removal reached 66%, compared with 59% for aliphatic.

The SDBS–Tween 80 mixtures exhibited clear differences in removal efficiency relative to SDBS alone when higher proportions of the non-ionic surfactant were used (25:75). Under these conditions, 62% of aliphatic and 72% of aromatic hydrocarbons were extracted. Aromatic removal exceeded aliphatic removal across all hydrocarbon size ranges except C8–C10 and C21–C35.

The results in Table 4 are consistent with the findings of Jousse et al. [64], who reported higher removal of aromatic than aliphatic compounds when using the non-ionic surfactant Tween 80. Similar trends were observed by Kuyukina et al. [79], who also achieved greater removal of aromatic hydrocarbons in crude-oil-contaminated soil. These authors attributed the lower removal of aliphatic compounds, particularly those with high molecular weight, to their stronger adsorption onto soil components, which reduces their desorption and subsequent extractability. No studies were found that evaluate hydrocarbon fractions in soils treated with mixed surfactants, preventing direct comparison with the present results. Nevertheless, previous research has reported hydrocarbon fractionation following treatment with individual (unmixed) surfactants.

The removal performance observed for each hydrocarbon group, according to both chemical structure and molecular size, can be interpreted in relation to the octanol–water partition coefficient (K_ow_) [82]. K_ow_ represents the ratio of a compound’s concentration in n-octanol to that in water in a biphasic system and therefore reflects its differential solubility in these two immiscible solvents. Compounds with high K_ow_ values are strongly hydrophobic, whereas those with low K_ow_ values are comparatively hydrophilic [83]. Petroleum hydrocarbons generally exhibit high K_ow_ values, which explains their limited affinity for the aqueous phase [84].

Aromatic hydrocarbons typically have lower K_ow_ values than aliphatic hydrocarbons [85], and the log K_ow_ values for the hydrocarbon fractions used in this study are presented in Table 5.

This property may account for the higher removal of aromatic compounds relative to aliphatic ones: lower K_ow_ values facilitate desorption from soil surfaces and enhance solubilisation within surfactant micelles. Conversely, K_ow_ increases with carbon number [85], which is consistent with the lower extraction efficiencies observed for higher-molecular-weight hydrocarbons (Figure 3 and Figure 4). Zhou & Zhu [45] indicated how the desorption of hydrophobic organic compounds from the soil by surfactants was related to an octanol–water partition coefficient as well. Zhou & Zhu [45] made a model that correlated the desorption of several PAHs with a non-ionic surfactant TX-100 solution with the K_ow_ of PAHs.

In addition, determining the residual concentrations of contaminants after treatment is essential to verify whether the remediated soil meets acceptable quality standards or whether additional treatment steps are required. Figure 4 presents the four major hydrocarbon fractions remaining in the soil after surfactant flushing. SDBS did not exhibit preferential removal, and the post-washing distribution of hydrocarbon fractions remained similar to the initial composition (aliphatic volatiles 22%, aliphatics 42%, aromatic volatiles 21%, and aromatics 15%).

In contrast, the SDBS–Brij 35 mixtures reduced the proportion of volatile compounds (C6–C10) and increased the relative abundance of aliphatic hydrocarbons (C10–C35) compared with SDBS alone. This effect became more pronounced as the proportion of Brij 35 in the mixture increased (Figure 4a).

For the SDBS–Tween 80 mixtures, the initial hydrocarbon distribution was largely maintained at ratios of 80:20 and 67:33. However, when the proportion of Tween 80 increased to 50:50 and 25:75, the fraction of volatile aliphatic C6–C10) decreased, while the proportion of heavier aliphatic hydrocarbons (C10–C35) increased (Figure 4b). These findings are consistent with Li et al. [80], who reported that Tween 20 exhibited limited effectiveness in removing heavier hydrocarbon compounds.

## 4. Conclusions

Surfactant-mixture ratios were evaluated to remediate soil contaminated with gasoline and diesel fuel. Hydrocarbon removal increased as surfactant concentration rose. However, the improvement in the removal efficiency obtained at concentrations higher than 1.5% was relatively minor compared to the additional surfactant required. The use of mixed surfactants enhanced hydrocarbon extraction compared to the individual surfactant. The mixtures exhibited different removal efficiencies depending on the proportions of anionic and non-ionic surfactants used. The most effective combinations were the SDBS:Brij 35 mixtures at 80:20, 67:33, and 50:50 ratios.

Regarding significance, a separate analysis of the different gasoline and diesel hydrocarbon fractions showed that: (1) smaller hydrocarbons desorbed more readily than larger ones, (2) aromatic hydrocarbons exhibited slightly higher desorption than aliphatic compounds, and (3) increasing the proportion of non-ionic surfactant in the mixtures reduced the amount of volatile hydrocarbons (C6–C10) remaining in the soil while increasing the proportion of residual aliphatic hydrocarbons (C10–C35). These variations in removal efficiency across hydrocarbon fractions may be related to their octanol–water partition coefficients.

This study confirmed that mixed anionic (SDBS) and non-ionic (Brij 35) surfactants have strong potential for application in soil and groundwater remediation. The findings provide valuable insights for improving the design of in situ remediation strategies for petroleum-hydrocarbon-contaminated soils through the use of surfactants. The use of mixed surfactants enhanced the efficiency of the soil-flushing system, making it a more cost-effective remediation approach.

From a regulatory standpoint, the enhancements accomplished through the utilisation of mixed-surfactant systems hold significance within the framework of European and national soil quality directives, which increasingly emphasise the need to reduce hydrocarbon concentrations to levels compatible with environmental protection and human health. Despite the divergent regulatory thresholds across Member States, numerous guidelines, including Spanish regional reference values, establish indicative limits for TPH fractions that often require substantial reductions before land can be reused or restored. The removal efficiencies obtained in this study suggest that mixed-surfactant formulations could assist in bringing contaminated soils closer to these regulatory benchmarks, thereby reinforcing the practical applicability of surfactant-assisted washing in real remediation scenarios.

Despite the fact that the present study did not directly evaluate bioremediation processes, the results indicate that surfactant-based washing may serve as a preparatory or complementary step within integrated remediation strategies. The partial desorption of diesel hydrocarbons achieved by surfactant mixtures could enhance the bioavailability of residual contaminants, a condition commonly exploited in subsequent bioremediation treatments. Consequently, the integration of surfactant washing with biological remediation, whether in a sequential or an integrated treatment scheme, is identified as a promising avenue for future research, particularly in the context of in situ applications within the regulatory frameworks.

Future studies might augment the present findings by deepening the mechanistic understanding of surfactant–soil interactions through the development of adsorption isotherms and the quantification of surfactant behaviour within the soil matrix. Concurrently, the exploration of biosurfactants as more sustainable alternatives, whether individually or in combination with synthetic surfactants, is a promising avenue for enhancing the environmental performance and long-term applicability of soil-washing technologies.

## Figures and Tables

**Table 1:** Soil properties.

Properties			
Particle size (%)	Sand	Silt	Clay
	20.6	58.2	21.2
Texture	Silt loam		
Organic matter (%)	5.1		
pH (pH units)	5.9		

**Table 2:** Initial hydrocarbon concentration (mg hydrocarbon/kg soil).

Initial Hydrocarbon Concentration (mg Hydrocarbon/kg Soil)							
Hydrocarbon size	C6–C8	C8–C10	C10–C12	C12–C16	C16–C21	C21–C35	C6–C35
Aliphatic	820	610	580	1300	1410	640	5360
Aromatic	690	870	570	390	510	140	3170
Total	1510	1480	1105	1690	1920	780	8530

**Table 3:** Surfactant mixtures.

Mixture	Mass/Mass Ratio (%)				
SDBS-Brij 35	100:0	80:20	67:33	50:50	25:75
SDBS-Tween 80	100:0	80:20	67:33	50:50	25:75

**Table 4:** Effect of surfactant mixtures on the aliphatic and aromatic fractions in gasoline and diesel fuel.

		SDBS	SDBS-Brij 35				SDBS-Tween 80			
		100:0	80:20	67:33	50:50	25:75	80:20	67:33	50:50	25:75
Aliphatic	C6–C8	65	80	80	80	83	64	61	77	77
	C8–C10	71	87	88	89	91	74	70	87	86
	C10–C12	62	72	69	67	59	67	64	67	69
	C12–C16	55	65	63	61	47	60	57	59	58
	C16–C21	50	58	57	55	42	54	51	51	48
	C21–C35	53	61	57	55	39	55	51	71	49
	Total	57	68	67	66	59	58	56	66	62
Aromatic	C6–C8	68	76	80	83	76	66	66	75	83
	C8–C10	61	74	75	76	75	62	62	71	75
	C10–C12	65	70	70	70	61	61	70	70	74
	C12–C16	62	69	69	69	59	59	66	62	72
	C16–C21	49	53	53	53	45	38	53	47	55
	C21–C35	50	25	42	53	40	31	50	41	45
	Total	61	68	70	71	66	57	62	65	72

**Table 5:** Log K_ow_ hydrocarbon fraction values.

	C6–C8	C8–C10	C10–C12	C12–C16	C16–C21	C21–C35
Aliphatic	3.78	4.76	5.74	7.22	9.18	13.6
Aromatic	2.43	3.15	3.72	4.46	5.61	7.28

## Data Availability

The original contributions presented in this study are included in the article. Further inquiries can be directed to the corresponding author.

## Nomenclature

CMC	Critical micelle concentration
COCs	Chlorinated organic compounds
K_ow_	Octanol–water partition coefficient
PAHs	Polycyclic aromatic hydrocarbons
SDBS	Sodium dodecyl benzenesulfonate
SDS	Sodium dodecyl sulfate
TPH	Total petroleum hydrocarbon
TX-100	Octylphenol polyethoxylate
